# Behavioural Change, Indoor Air Pollution and Child Respiratory Health in Developing Countries: A Review

**DOI:** 10.3390/ijerph110504607

**Published:** 2014-04-25

**Authors:** Brendon R. Barnes

**Affiliations:** Psychology Department, University of Johannesburg, Auckland Park, 2006, South Africa; E-Mail: bbarnes@uj.ac.za; Tel.: +27-824-487-782

**Keywords:** behavioural change, indoor air pollution, household energy, theory

## Abstract

Indoor air pollution caused by the indoor burning of solid biomass fuels has been associated with Acute Respiratory Infections such as pneumonia amongst children of less than five years of age. Behavioural change interventions have been identified as a potential strategy to reduce child indoor air pollution exposure, yet very little is known about the impact of behavioural change interventions to reduce indoor air pollution. Even less is known about how behaviour change theory has been incorporated into indoor air pollution behaviour change interventions. A review of published studies spanning 1983–2013 suggests that behavioural change strategies have the potential to reduce indoor air pollution exposure by 20%–98% in laboratory settings and 31%–94% in field settings. However, the evidence is: (1) based on studies that are methodologically weak; and (2) have little or no underlying theory. The paper concludes with a call for more rigorous studies to evaluate the role of behavioural change strategies (with or without improved technologies) to reduce indoor air pollution exposure in developing countries as well as interventions that draw more strongly on existing behavioural change theory and practice.

## 1. Introduction

Approximately half the world’s population are reliant on biomass fuels such as wood, cow dung and crop residues for their domestic energy requirements [1]. The indoor burning of biomass fuel releases smoke that contains numerous pollutants such as carbon monoxide (CO), particulate matter (PM) and other organic compounds into the living environment [2]. Estimates indicate that indoor air pollution is associated with 1.5 million deaths annually and 2.7% of the global burden of disease [3]. Of particular concern is the association between indoor air pollution and child Acute Respiratory Infections (ARI) [4].

Children under five years old are considered to be susceptible to ARI through indoor air pollution exposure for a number of reasons. The epithelial linings of children’s lungs are not fully developed resulting in greater permeability of pollutants [5], their immune systems are not fully developed thereby limiting the body’s defence against infection [6], they have higher respiration rates, narrower airways and they have a larger lung surface area per kilogram of body weight thus breathing in approximately 50% more (polluted) air under normal breathing conditions compared to adults [7]. In addition, children tend to spend extended periods of time in the vicinity of indoor fires during peak cooking and heating times [8,9].

Given the burden of child ARI, the high reliance on biomass fuels in developing countries, and the strength of the evidence associating indoor air pollution with child ARI [10,11,12], urgent calls have been made for interventions to reduce child exposure to indoor air pollution. A number of prevention interventions have been identified to reduce child exposure to indoor air pollution [13] including technical interventions such as improved cook stoves and improving access to cleaner burning fuels. Improved cook stoves have shown to significantly reduce indoor air pollution [14] and have understandably received most of the attention.

As scientific attention turned towards evaluating the health benefits of technical indoor air pollution interventions, the field was also beginning to understand the behavioural determinants of indoor air pollution exposure [15]. Behaviours—for example, where people burn fires, how fuels are dried, how fires are kindled, how people open and close windows and where children are located in relation to indoor fires—may account for the wide range of exposure estimates documented from households with similar energy patterns (for example, fuels and stove types) and ventilation characteristics [16]. Behavioural change strategies targeting these behaviours may reduce indoor air pollution exposure.

In addition, behavioural interventions may also play an important role in supporting improved cook stove programs by creating the demand, promoting the correct use and maintenance of appliances as well as improving their sustainability. A study in China [17] (reviewed in more detail below), for example, showed that indoor air pollution interventions could be maximised by focusing on both improved cooking technologies combined with behavioural change. *Similar to technical interventions, however, very little is known about the impact of behavioural change on both indoor air pollution exposure and child ARI* [13].

Behavioural change interventions in environmental health are notoriously difficult [18] and reviews of the published evidence have shown that they fail far more often than they succeed [19]. Behavioural change theory plays a critical role in guiding how interventions are conceptualised and research has shown that behavioural interventions informed by theory tend to be more successful than those that are not [20]. By the very least, it is important that intervention programs include underlying program theory or a theory of change to understand why behavioural interventions work or not.

A dominant theme in the indoor air pollution/behaviour change literature is that health education may be sufficient to elicit behavioural change. This is reflected by the many calls for more public health awareness [21], education programs [22], parental education [23], maternal education [24] and so forth to reduce indoor air pollution exposure at the household level. These calls usually appear in the conclusion section of articles and health education is usually represented as a ‘simple’ alternative to more expensive technological interventions. Yet, we know from the broader behavioural change literature that educating people about health risks may not be sufficient for behavioural change [25].

Behavioural scientists and practitioners have a range of theoretical models to assist them with planning the content of interventions and deciding at what level(s) interventions are pitched. These include models such as the Health Belief Model, the Theories of Reasoned Action and Planned Behaviour, Protection Motivation Theory, Social Learning Theory, Applied Behavioural Analysis, the Trans theoretical Model, Diffusion of Innovation, Social Marketing and so forth [26]. Each of these theories offers a more nuanced picture of human behaviour than knowledge of health risks.

For example, if framed by the Health Belief Model (one of the most commonly used health belief theories worldwide), a behavioural intervention would not be limited to imparting health information but would also include the options of improving the perceptions of the seriousness of the illness (children can die from lower respiratory infections) and perceptions of susceptibility (even short periods of exposure might be harmful to the child). The intervention could also work with families to improve the skills needed for a successful intervention, enhance cooks’ confidence to undertake the behavioural recommendations, emphasize the potential health benefits, cost savings and possibly the social status associated with the program while taking into account social, cultural and other potential barriers. It is beyond the scope of this paper to cover each of the theories listed above, suffice to say that they each offer a more complex intervention strategy than health education. *Yet, it is unclear if/how behavioural change theory has been incorporated into indoor air pollution and behavioural change intervention studies beyond calls for more health education.*

In response to these gaps, this review was driven by two interrelated questions: what are the potential impacts of behavioural interventions on indoor air pollution exposure? How has behavioural theory been incorporated (if at all) into behavioural change and indoor air pollution field studies?

## 2. Methodology

The following databases were searched for peer reviewed published journal articles: PUBMED, MEDLINE, PsycLIT, PSYCINFO, ERIC, Google Scholar and Science Direct to obtain an initial pool of research articles. In addition, Web of Science as well as the Cochrane Library were searched to identify risk specific systematic reviews and meta-analyses. English language papers for the period 1983–2013 (30 years) were included. MeSH terms included: developing countries, indoor air pollution, household air pollution, behavioural change, behavioural modification, behavioural change communication, health promotion, household energy, quantitative, qualitative and theory. Studies had to include or model the impact of behavioural change on indoor air quality or child respiratory health outcomes (although just one study fulfilled the latter criteria). Thus even though several behavioural change and indoor air pollution studies existed, they were excluded from the analysis if they did not measure indoor air pollution or child respiratory health. A total of two cross sectional, five laboratory and three intervention studies were identified and included in the review. Field studies were reviewed for evidence of behavioural theory as laboratory studies typically do not include behavioural theory.

## 3. Results

Studies (summarized in Table 1 and Table 2) have highlighted indoor air pollution reductions of between 20%-98%. The results are divided into laboratory studies and field studies for ease of reporting.

### 3.1. Laboratory Studies

A study [27] in South Africa focused on the impact of an alternative ignition process for coal fires on indoor air quality. The study reported that the upside-down ignition method in a commonly used burning appliance (the brazier or “mbawula”)—where fires are ignited with coal at the bottom, paper/wood kindle above the coal and a small amount of coal on top (instead of the regular method of paper/wood kindle at the bottom and coal at the top)—reduced PM_10_ by 80%–90% in laboratory testing (actual indoor air pollution levels, however, were not reported) and by approximately 50% under actual field testing [27]. This behavioural strategy has been included in South Africa’s Integrated Household Clean Energy Strategy [27].

Other studies have focused on the role of tending fires in relation to indoor air pollution. Laboratory studies in the early 1980s showed that well-tended fires display much higher levels of efficiency and lower emission characteristics than previously thought, and in some cases are comparable to efficiency figures obtained from improved cook stoves ([28] cited in [29]). Behaviours such as using smaller stones to hold the pot snugly, tending the fire with shorter pieces of wood that are kept constantly under the pot, the use of dry wood of uniform sizes and the use of pot lids may all improve the efficiency and emissions of wood fires [29]. Similarly a study [30] found that compared to an open fire, improving an open fire by burning fuels on a raised grate 10mm off the ground was associated with 20% lower TSP (total suspended particulates) and 41% lower CO emissions compared to a wood fire burned on the ground and comparable, and sometimes better than, emissions from improved cook stoves per burning.

Two studies have shown the potential of opening ventilation during indoor burning. A laboratory study showed notable reductions through simply opening a door during burning [31]. Under controlled laboratory conditions, the study found that opening a door for the duration of burning showed 94% lower PM_10_ and 97% lower CO compared to concentrations measured in an unventilated closed kitchen. Similarly, Grabow, Still and Benson [32] measured indoor air pollution in a laboratory kitchen of approximately 24 m^3^ using a coal burning test stove. Compared to a closed kitchen with little air exchange, results showed that opening the door and window during burning reduced one hour concentrations of PM_10_ by between 93% and 98% and one hour concentration of CO by between 83% and 95% [33].

**Table 1:** Laboratory studies.

Study	Target Behaviours	Fuel Type	Indoor Air Pollution Impact
Surridge *et al*., (2005) [27] Pretoria, South Africa	Reverse ignition process for coal fires	Coal	Reduced PM_10_ by 80%–90% in laboratory testing (indoor air pollution levels, however, were not reported) and by approximately 50% under actual field testing
Bussman, Visser and Prasad (1983) [28] cited in Manibog (1984) [29] India	Ensure pot fits snugly over fires Use pot lids Use smaller pieces of wood of uniform sizes Dry wood	Wood	77% CO and 94% PM reduction associated with these behaviours
Ballard-Tremeer and Jawurek (1996) [30] Johannesburg, South Africa	Raise grate 10 mm off the ground	Wood	20% lower TSP (total suspended particulates) and 41% lower CO emissions compared to a wood fire burned on the ground
Still and MacCarty, (2006) [31] Oregan, USA	Open windows and doors during cooking	Charcoal	94% lower (1 h) PM and 97% lower (1 h) CO compared to concentrations measured in an unventilated closed kitchen
Grabow, Still and Benson (2013) [32] Oregan, USA	Open windows and doors during cooking	Coal	Compared to closed ventilation, (1 h) PM reduced by 93%–98% and (1 h) CO by 83%–95%

**Table 2:** Field studies.

Study	Study Design	Target Behaviours	Fuel Type	Indoor Air Pollution Impact	Evidence of Behaviour Change Theory
Reid, Smith and Sherchand, (1986) [33] Nepal	Cross sectional. Indoor air pollution monitored in 60 households with observations of cook behaviours	Use and maintain cooking appliances	Wood	PM reduced by over 75% (from 4900 to 1100 μg/ m^3^) and CO by 94% (from 500 to 31 parts per million (ppm)) when correct fitting pots were used. Cleaning stove flues reduced CO from 500 to 56 ppm	No
Albalak (1999) [34] Bolivia	Cross sectional. A total of 621 IAP measurements taken from Bolivian households	Burning location: indoor *versus* outdoor	Variety of biomass fuels	PM_10_ exposure for infants estimated to be 63% higher (15,360 μg/h/m^3^ for the indoor cooking village compared to the outdoor cooking village (5760 μg/h/m^3^)	No
Jin *et al*., (2006) [17] China	Field trial. 25–30 households monitored in each condition and across provinces	Health education and behavioural activities (HEBA) together with improved cook stoves in four rural provinces in China. Exact target behaviours are unclear	Coal and a variety of biomass	Up to 85% reduction in selected indicators	No
Tun *et al*., (2005) [35] Tibet	Quasi experiment field trial with comparison group. 331 under-fives in the intervention group and 338 in the control group were followed for 6 months. ARI incidence measured but not IAP	Focused on the causes and prevention of ARI with special emphasis on the avoidance of indoor air pollution	Unclear but biomass, mosquito coils and scented sticks were referred to in the article	Not measured/reported	No
Barnes, Mathee & Thomas (2011) [36] South Africa	Quasi experiment field trial with comparison group. *n* = 36 households and children (youngest in the family) in the intervention and *n* = 38 households in the control group. IAP measured before and after the intervention.	Community counselling but with a strong focus on burning outdoors and opening windows and doors when cooking indoors	Wood, dried cow dung and coal.	Net median reductions were PM_10_ = 57%, CO = 31% and CO (child) = 33% amongst households that burned indoor fires	No formal theory but factors described in Barnes (2010) [37]

### 3.2. Field Studies

The maintenance and proper use of cooking appliances may also impact on the emissions of those appliances. A study in Nepal [33] focused on poor pot fit and poor flue cleaning of improved cook stoves in relation to indoor air pollution. The study found that particulate matter was reduced from 4900 to 1100 μg/m^3^ and CO from 500 to 31 ppm when correct fitting pots were used in improved cook stoves. The study also found that cleaning stove flues (by removing 1.5 litres of soot) reduced CO from 500 to 56 ppm (89%) [33].

A cross-sectional study in rural Bolivia [34], for example, measured indoor air pollution exposure in two similar villages—one where cooking was done indoors and one in which cooking was primarily done outdoors. The villages were similar in terms of socio-demographic, cultural and climatic conditions. The only difference was behavioural: cooking was done in small kitchens with very little ventilation in the “indoor cooking village” while cooking was done primarily outdoors in an area defined by semi-circular wall made of root plant in the “outdoor cooking village”. Monitoring of PM_10_ was done in three locations in both villages: the home, the kitchen and outdoors. Amongst others, results showed significant differences in personal exposures between the two villages. Estimated daily PM_10_ exposure for infants during the non-work season (when people tend to spend more time indoors) were estimated to be three times higher (15,360 μg/h/m^3^ derived from stationary levels of PM_10_ divided by the reported time to be in the vicinity of fires) for the indoor cooking village compared to the outdoor cooking village (5760 μg/h/m^3^) [34].There was no evidence of behavioural change theory.

A quasi-experimental intervention study monitored ARI incidence for six months in 331 children under five years old in an intervention and 338 children in a control community in Yangdon, Tibet [35]. Baseline data on maternal knowledge, attitudes and indoor air pollution practices were collected once before and once six months after the health education intervention. The key messages are not well described in the article but the health education reportedly focused on “the causes and prevention of ARI with special emphasis on the avoidance of indoor air pollution” (p. 31). Mothers were visited once to explain the intervention and were offered pamphlets. Wall posters were placed in the market place, tea shops and local authority offices [35]. The study found at follow-up that although caregivers knowledge of indoor air pollution was significantly increased amongst the intervention group (compared to the control group), there was no significant differences between the two groups on location of cooking (in living room, kitchen or outside), type of fuel used or mosquito deterrent behaviours (use of scented sticks and/or coils). There was also no impact on ARI incidence, which increased in both groups following the intervention. No explicit behavioural theory was evident.

A comprehensive Chinese behavioural trial [17] tested the effectiveness of ‘health education and behavioural activities’ (HEBA) together with improved cook stoves in four rural provinces in China (Gansu, Guizhou, Shaanxi and Inner Mongolia). Study populations in each of the provinces were divided into three groups at the township level: one group received improved cook stoves together ‘health education and behavioural activities’ (HEBA), the second group received only HEBA without the technology while the third group received no intervention (control group). Extensive baseline monitoring including knowledge, practices, indoor air pollution exposure (PM_10_, CO and SO_2_) and health outcomes were conducted before the intervention [17].

The HEBA implementation involved the following steps in all provinces: (1) explain the source of indoor air pollution; (2) explain the health hazards of exposure; (3) explain the benefits of fuel, stoves and ventilation improvements; and (4) alternative stove use behaviour. Results suggest that although there were incremental increases in knowledge of indoor air pollution, the HEBA on its own showed no impact on indoor air pollution exposure (PM_10_, CO and SO_2_). The combination of HEBA with improved stoves showed measurable improvements in indoor air quality (by as much as 85%) and efficiency. Although the study utilised a relatively rigorous trial methodology, no theory was reported in the intervention.

A quasi experimental study (with a comparison group) [36] used a community counselling approach to encourage caregivers to reduce their children’s indoor air pollution exposure in rural villages in South Africa. Although the study focused on improving a number of behaviours, the intervention had a strong focus on opening windows and doors at strategic times during the burning process, for example, during ignition or when wood was added to the fires. The study evaluated the impact of the intervention on stationary levels of PM_10_ and CO as well as CO measured on children younger than five. Using a quasi-experimental design, baseline indoor air quality data were collected in an intervention (*n* = 36) and a comparison (*n* = 38) community; the intervention was implemented in the intervention community only during winter when indoor cooking and heating is common; and follow-up data were collected one year later amongst the same households. Despite the fact that indoor air pollution was reduced in both communities (possibly due to a Hawthorne Effect) the intervention group performed significantly better than the control group among those who cooked indoors. The net median reductions associated with the intervention were: PM_10_ = 57%, CO = 31% and CO (child) = 33% amongst households that burned indoor fires. No explicit theory was evident.

However, a qualitative follow-up study of the above intervention aimed to understand *why* participants improved their behaviours [37] to develop a theory of change. The study found that although improved health perceptions played a role (especially specific perceptions such as perceptions of susceptibility), other factors such as reduced drudgery (cooking outdoors made indoor cleaning easier) and social standing (those who cooked indoors were often deemed as backward) also played an important role. Barriers to behavioural change included the need for warmth during winter, perceptions that indoor air pollution is a normal part of rural existence and gender (men made it difficult for women to comply with behavioural recommendations) [37].

## 4. Discussion

It is relatively clear that behavioural change (with or without improved technology) offers the potential to reduce child exposure to indoor air pollution. However, similar to other indoor air pollution interventions [38], the evidence is based on a limited number of studies (just 10 studies over a period of 30 years with just three of these being intervention studies) with several methodological shortcomings. First, except for the three intervention studies, much of the evidence base is limited to cross-sectional studies and laboratory studies. Cross-sectional studies offer little information about the situation before the intervention was introduced, why the intervention was sustained in certain contexts (and not in others) and the effects of the intervention over time.

Second, there has been a lack of consistency in the way indoor air pollution exposure has been measured. Studies have focused on a variety of pollutants (for example, particulate matter, carbon monoxide or a combination) using different monitoring equipment. Studies have also differed in their methodological approach to indoor air pollution monitoring. For example, in measuring particulate matter, some studies have used a time-weighted average approach while other studies have used real-time monitoring. Studies have also differed in their monitoring periods (e.g., duration of a burning fire, 1 h, 8 h or 24 h). Importantly not many studies have measured personal exposure and just one study attempted to measure health outcomes [35]. This makes comparing effects across studies difficult to achieve.

Formal behaviour change theory was non-existent in the literature reviewed and just one study described the factors that influenced behavioural change. Of particular importance to this review was the question of whether messages aimed at educating caregivers of the *health* risks of indoor air pollution was associated with the uptake of improved behaviours at the household level. A post trial qualitative study in South Africa [37] found that although improved health perceptions played a role, other factors such as perceptions of social standing, gender, cold weather and drudgery also played a role. Similarly, the Chinese [17] and the Tibetan [35] trials showed limited effectiveness of health education on its own (although it should be noted that the interventions themselves were not well described). This review confirms an important point conveyed in the broader health and behavioural change literature [39]—that improving health considerations alone may have a relatively minor contribution to sustained behavioural change. Because health behaviour change models have been poorly integrated into the indoor air pollution literature, it is very difficult to suggest which of the existing models are appropriate. Nonetheless, models do exist and could be adapted to local contexts. However, much more research is needed in this regard.

## 5. Conclusions

The study found reductions associated with behavioural change of 20%–98% in laboratory settings and 31%–94% in field settings. However, the evidence to support this is weak. There was also very little evidence of behavioural change theory in the field studies. More methodologically rigorous studies are needed to understand the impact of behaviour change on child indoor air pollution exposure and respiratory health. In addition, more theoretically informed work needs to be done to understand how and why participants may engage in protective behaviours to reduce child exposure to indoor air pollution.
